# Cupric Oxide Nanoparticles Induce Cellular Toxicity in Liver and Intestine Cell Lines

**DOI:** 10.34172/apb.2020.025

**Published:** 2020-02-18

**Authors:** Mahmoud Abudayyak, Elif Guzel, Gül Özhan

**Affiliations:** ^1^Department of Pharmaceutical Toxicology, Faculty of Pharmacy, Istanbul University, Istanbul, Turkey.; ^2^Department of Pharmaceutical Toxicology, Faculty of Pharmacy, Karadeniz Technical University, Trabzon, Turkey.; ^3^Department of Histology and Embryology, Cerrahpasa Medical Faculty, Istanbul University, Istanbul, Turkey.

**Keywords:** Copper oxide, Nanoparticle, DNA damage, Cytotoxicity, Oxidative stress, Apoptosis

## Abstract

***Purpose:*** The wide application of cupric oxide nanoparticles (copper (II) oxide, CuO-NPs) in various fields has increased exposure to the kind of active nanomaterials, which can cause negative effects on human and environment health. Although CuO-NPs were reported to be harmful to human, there is still a lack information related to their toxic potentials. In the present study, the toxic potentials of CuO-NPs were evaluated in the liver (HepG2 hepatocarcinoma) and intestine (Caco-2 colorectal adenocarcinoma) cells.

***Methods:*** After the characterization of particles, cellular uptake and morphological changes were determined. The potential of cytotoxic, genotoxic, oxidative and apoptotic damage was investigated with several *in vitro * assays.

***Results:*** The average size of the nanoparticles was 34.9 nm, about 2%-5% of the exposure dose was detected in the cells and mainly accumulated in different organelles, causing oxidative stress, cell damages, and death. The IC50 values were 10.90 and 10.04 µg/mL by MTT assay, and 12.19 and 12.06 µg/mL by neutral red uptake (NRU) assay, in HepG2 and Caco-2 cells respectively. Apoptosis assumes to the main cell death pathway; the apoptosis percentages were 52.9% in HepG2 and 45.5% in Caco-2 cells. Comet assay result shows that the highest exposure concentration (20 µg/mL) causes tail intensities about 9.6 and 41.8%, in HepG2 and Caco-2 cells, respectively.

***Conclusion:*** CuO-NPs were found to cause significant cytotoxicity, genotoxicity, and oxidative and apoptotic effects in both cell lines. Indeed, CuO-NPs could be dangerous to human health even if their toxic mechanisms should be elucidated with further studies.

## Introduction

People expose hazardously to nanoparticles – either anthropologically or as outputs of natural phenomena – via food, water, or air due to the gradual increases in nanomaterial usage in various aspects of life. Researches discourse that nanoparticles could be detected in the organs/tissues, brain, heart, liver etc.^1^ Similarly, copper (Cu)-based nanoparticles (NPs) were found in liver, kidney, and spleen following oral exposure.^2^ CuO-NPs are widely used in gas sensors, catalysts, high-temperature conductors, solar energy converters, and antimicrobial agents in industry, cosmetics, and medicine owing to their high conductivity, electron correlation effects, and special physicochemical properties.^3,4^ CuO-NPs caused morphological changes, necrosis, and dysfunction in liver, stomach, and kidney, disruption of the epithelial lining of the gastrointestinal tract and severe atrophy and color change in the spleen.^2,5-9^ CuO-NPs caused acute death, abnormalities, and damage in embryo and gill of Zebrafish.^10^ Generally, researchers have been interested in the toxic potentials of CuO-NPs on lung, skin, breast, brain and nervous system.^11-15^ However, there are only a few reports on liver and intestine cells.^16-19^ We comprehensively assessed the toxic potentials and toxicity mechanisms of CuO-NPs on HepG2 liver and Caco-2 intestinal cell lines. Their cytotoxic, genotoxic, oxidative damage, and apoptosis-necrosis induction potentials were investigated *in vitro* conditions following NPs characterization and evaluation of their cellular uptake. HepG2 and Caco-2 cell lines are highly differentiated and display many features of the liver and intestinal cells. Many researchers select these human cell lines as models of *in vitro* conditions to study the apical uptake, metabolism, and absorption of nutrients, chemicals and drugs.^20-22^

## Materials and Methods

### Chemicals

The materials and chemicals for cell culture as cell culture mediums (Eagle’s minimum essential medium [EMEM] and Dulbecco’s modified eagle medium [DMEM]), fetal bovine serum (FBS), phosphate-buffered saline (PBS X10), hydrogen peroxide (H_2_O_2_), Trypsin solution, and antibiotic-antimycotic solution were purchased from Multicell Wisent (Quebec, Canada). CuO-NPs, neutral red dye (NR), ethylenediaminetetraacetic acid (EDTA), dimethyl sulfoxide (DMSO), triton X-100, glacial acetic acid, and MTT (3-[4,5-dimethylthiazol-2-yl]-2,5-diphenyl-tetrazolium bromide) were from Sigma Chemical Co. Ltd. (St. Louis, MO, USA). Glutathione (GSH), 8-hydroxy deoxyguanosine (8-OHdG), malondialdehyde (MDA) and protein carbonyl (PC) enzyme-linked immune sorbent assay (ELISA) kits were from YEHUA Biological Technology Co., Ltd. (Shanghai, China). Annexin V-FITC apoptosis detection kit with propidium iodide (PI) and dye reagents for protein assay was from Biolegend (San Diego, CA, USA) and Bio-Rad (Munich, Germany), respectively.

### Particle size characterization

CuO-NPs were suspended in Milli-Q water and cell culture medium with 10% FBS and measured by transmission electron microscopy (TEM) (JEM-2100 HR, JEOL, USA).^23,24^ The average hydrodynamic size was determined by dynamic light scattering (ZetaSizer Nano-ZS, Malvern, UK) in the cell culture medium.

### Cu’s release into cell medium and cellular uptake

The cellular uptake of nanoparticles and Cu release to the medium were determined by inductively coupled plasma-mass spectrometry (ICP-MS) (Thermo Elemental X series 2, USA). For that, the exposed cells were harvested and counted, after that cells were digested by treatment with nitric acid for 6 hours in room temperature.^23,24^

### Cell culture conditions

Human HepG2 hepatocarcinoma (HB-8065; American Type Culture Collection [ATCC] Rockville, MD, USA) and Caco-2 colorectal adenocarcinoma (HTB-37; ATCC, Rockville, MD, USA) cells were used according to the manufacturer’s instructions. The cell densities were in the range of 1×10^4^- 1×10^7^ cells/mL. The exposure time was 24 h.

### Cellular uptake and morphology examinations by TEM

TEM measurements were used for both uptake and morphological changes evaluation.^23,24^ For this, ultra-thin sections (50-60 nm) of the exposed cells were cut by an ultramicrotome (Reichert UM 3, Austria). Sections were analyzed by a TEM (Jeol-1011, Tokyo, Japan) with an attached digital camera (Olympus-Veleta TEM Camera, Tokyo, Japan).

### Cytotoxicity

The cytotoxic potential was determined by MTT and neutral red uptake (NRU) assays.^23-24^ The exposure concentrations were in the range of 2.5-60 µg/mL. Triton X-100 (1%, v/v) was used as a positive control (PC). Optical density (OD) values were read by a microplate spectrophotometer system (Epoch, Germany). The inhibition of enzyme activity or the uptake of pigment observed in cells was compared to the negative control (NC). The half-maximal inhibitory concentration (IC_50_) was expressed as the concentration of sample causing a 50% inhibition of enzyme activity in cells.

### Genotoxicity

The genotoxic potential was determined by comet assay.^23,24^ The exposure concentrations were in the range of 5-20 µg/mL. At the highest concentration, cell death was ≤ 50%. H_2_O_2_ (100 µM) and PBS 1X were used as PC and NC, respectively. The degrees of deoxyribonucleic acid (DNA) breaks’ were scored under a fluorescent microscope (Olympus BX53, Tokyo, Japan) at 400X using an automated image analysis system (Comet Assay IV, Perceptive Instruments, Suffolk, UK). DNA damage in individual cells was expressed as a percentage of DNA in the comet tail intensity.^25^

### Oxidative damage

The oxidative damage was evaluated by assessing the cellular levels of GSH, MDA, 8-OHdG, and PC parameters. For that, human ELISA kits according to the manufacturer’s instructions. Briefly, the cells were collected and washed with cold BPS 1X, raptured by repeated frozen-thaw process, centrifuged at 2500 revolutions per minute (rpm) for 20 minutes, 40 µL of the supernatant of the cells were added to the wells containing monoclonal antibody, 10 µL of Biotin-labelled antibody, and 50 µL of streptavidin-HRP were added. After incubation for 1 h at 37°C, 50 µL of chromogenic reagent A and 50 µL of chromogenic reagent B were added and incubated for farther 10 minutes. 50 µL of the stop solution was added. The OD values were read at 450 nm by a microplate spectrophotometer system (Epoch, Germany). The exposure concentrations were in the range of 5-25 µg/mL. The unexposed cells were evaluated as NC group. The protein amount in 10^6^ cells was measured according to Bradford’s method.^26^ The results were expressed for g of protein using a standard calibration curve.

### Apoptosis

An Annexin V-FITC apoptosis detection kit with PI was used.^23,24^ The exposure concentrations were in the range of 10-80 µg/mL. The exposed cells were unattached, washed twice with PBS, after centrifuging for 3 minutes at 1200 rpm cells were suspended in PBS to be 10^6^ cells/ mL. 100 µL of cell suspension was treated with 5 µL of annexin v and 5 µL of PI dyes and incubated for 15 minutes at the darkness in room temperature. Dyed cells were dropped on slides and covered with slips. Green cells were accepted as apoptotic, red ones as necrotic and clear ones as viable cells. At least 1000 cells for each sample were counted. The results were expressed as a percentage of the total cell amount. The unexposed cells and cells incubated at 56°C for 30 minutes used as NC and PC, respectively.

### Statistical analysis

The assays were done in triplicate and each assay was repeated twice. Data were expressed as mean ± standard deviation (SD). Significant differences between untreated and treated cells were calculated by one-way ANOVA and post hoc Dunnett *t* test using SPSS version 23.0 for Windows (SPSS Inc., Chicago, IL).*P* values of less than 0.05 were considered significant.

## Results

### Particle features

**Figure 1:**
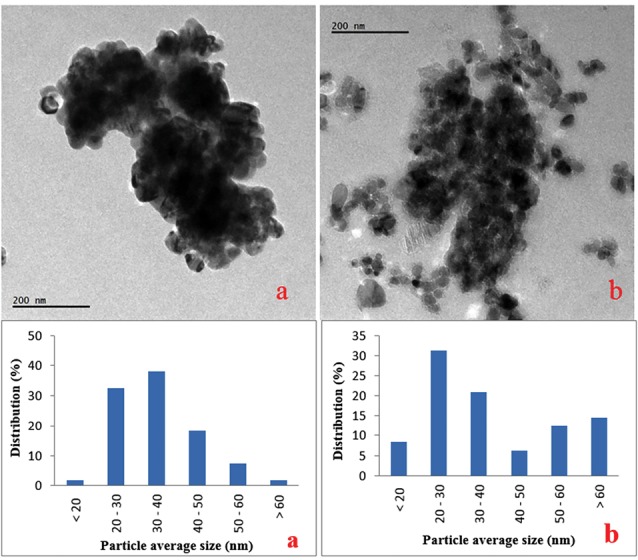
TEM images and size distributions of CuO-NPs in water (a) and cell culture medium (b).

**Figure 2:**
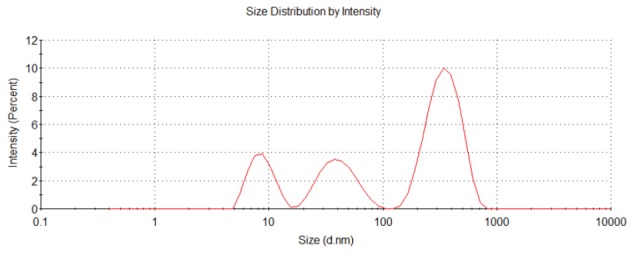
CuO-NPs characterisation by dynamic light scattering in the cell culture medium.

According to their manufacturer (Sigma Chemical Co. Ltd., USA), the particle size of the CuO-NPs was ≥ 50 nm, the surface area was 29 m^2^/g, and the X-ray diffraction results confirm to the structure of the particles. Our results confirmed that; the average size was 34.9 nm (ranging from 16.7 - 64.2 nm) after suspending in water. The particles were slightly agglomerated/aggregated in medium (38.8 nm ranging from 18.8 - 73.8 nm) (Figure 1). CuO-NPs hydrodynamic size was mean 221.53 nm (8.29-342.13 nm) with 40% of the particles has a size lower than 38 nm (Figure 2).

### Cu release into cell medium and cellular uptake

The ICP-MS assay results show that Cu ions were not detected in cell-free medium, while 2%-5% of the exposure dose was detected in the cells, which indicates the uptake of CuO-NPs by HepG2 and Caco-2 cells following exposure for 24 h (Table 1). The differences observed in cellular uptake could be due to the differences in the permeability of the cells or due to agglomeration/aggregation of the nanoparticles in the high FBS cell medium (20%) used in Caco-2 cell line. Cu content of the unexposed cell (NC) was also measured for every cell line.

**Table 1:** Evaluation of the cellular uptakes of CuO-NPs

**Cells**	**Exposure concentration** **(µg/mL/10** ^ 5 ^ **cells)**	**Cu amount (ng/10** ^ 5 ^ **cells)**
HepG2	Negative control	140 ± 1.1
	10	356 ± 2.1
	25	528 ± 3.2
Caco-2	Negative control	148 ± 0.82
	10	302 ± 4.4
	25	289 ± 2.9

### Cellular morphology and uptake by TEM

**Figure 3:**
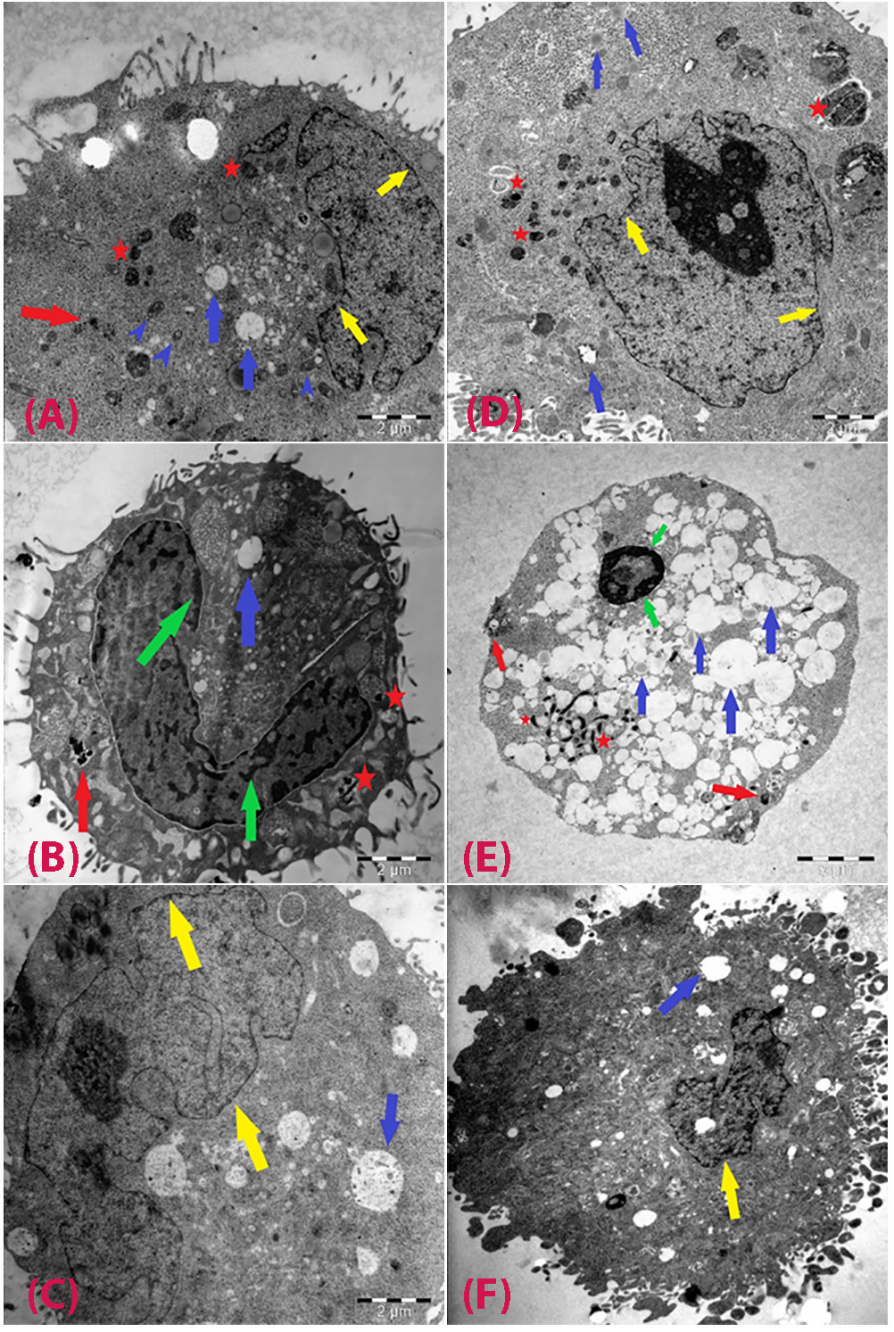
TEM observations of cells after exposure to CuO-NPs.
HepG2 cells exposed to 2.5 µg/mL (A), 10 µg/mL (B), and negative control (C); and Caco-2 cells exposed to at 2.5 µg/mL (D), 10 µg/mL (E), and negative control (F). Red arrows: CuO-NPs; blue arrows: vacuoles; green arrows: condensed chromatin; yellow arrows: intact plasma membranes; red star: particle containing vacuoles; blue arrow heads: healthy mitochondria.

The cellular uptake of particles and cell morphology were examined by TEM in both cells exposed to CuO-NPs and unexposed (NC) at 2.5 and 10 µg/mL (Figure 3). In HepG2 cells, mitochondria were visible, and an increase in the number of lipid droplets and cytoplasmic vacuoles was detected. Particle uptake increased depending on exposure concentration (Figure 3A, 3B, 3C). Caco-2 cells were observed low uptake level. Nanoparticle uptake into Caco-2 cells did not change when the exposure concentration was increased to 10 µg/mL. Electron-dense bodies were visible in the cytoplasm of most cells exposed to CuO-NPs. The nanoparticles were found within the electron-lucent vacuoles, and agglomeration/aggregation of the particles was observed in some of the vacuoles. Among the cells exposed to 2.5 µg/mL CuO-NPs, some exhibited abnormal nuclei with chromatin condensation and indentations of the nuclear membrane, and a few of the cells exhibited cytoplasmic fragmentation. Cellular damage increased with increasing doses of CuO-NPs. Many apoptotic and necrotic cells were detected (Figure 3 D, 3E, 3F).

### Cytotoxicity

**Figure 4:**
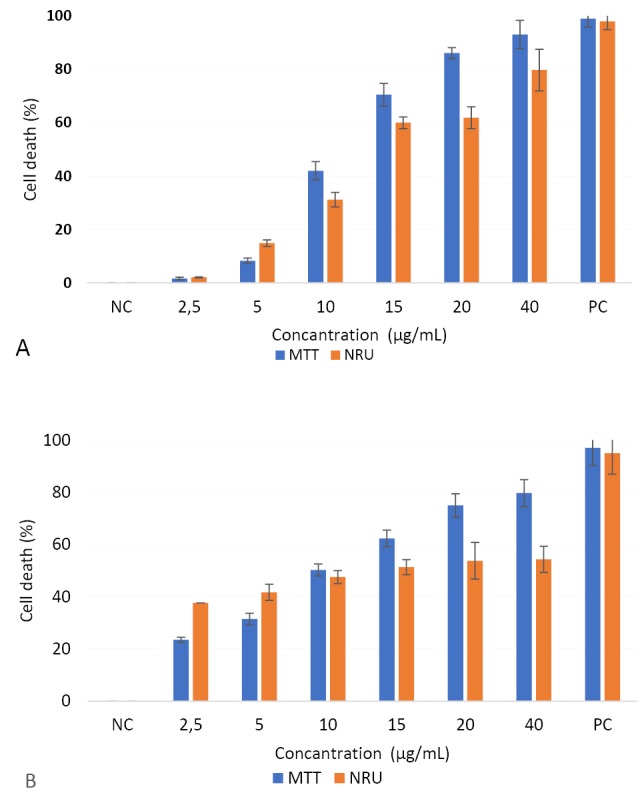
Effects of CuO-NPs on cell viability of HepG2 (A) and Caco-2 (B) cell lines.
The results were expressed as the mean cell death (%) compared to the negative control (NC, unexposed cell). Triton 100X (1%) was used as a positive control (PC).

CuO-NPs caused a reduction in the cellular metabolic activity and disruption in HepG2 and Caco-2 cells depending dose (Figure 4). IC_50_ values of CuO-NPs in HepG2 and Caco-2 cells were 10.90 ± 1.72 and 10.04 ± 0.56 µg/mL by MTT assay, and 12.2 ± 1.4 and 12.06 ± 0.83 µg/mL by NRU assay, respectively. Cellular sensitivity to cytotoxic damage induced by CuO-NPs was similar for the two cell lines.

### Genotoxicity

**Figure 5:**
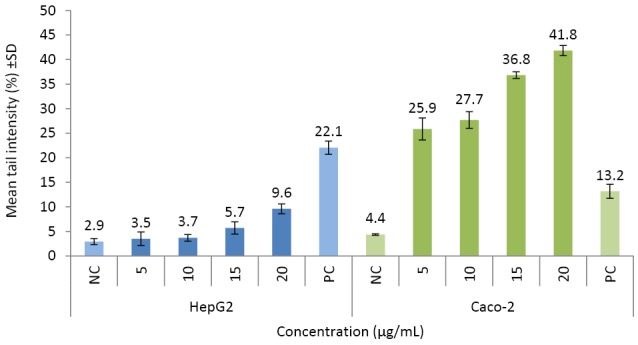
Evaluation of DNA damage potentials of CuO-NPs.
The results were presented as mean tail intensity (%) with ±SD. NC and PC refer to negative (unexposed) and positive (100 µM H_2_O_2_) controls, respectively. *P ≤ 0.05 were selected as the levels of significance.

**Figure 6:**
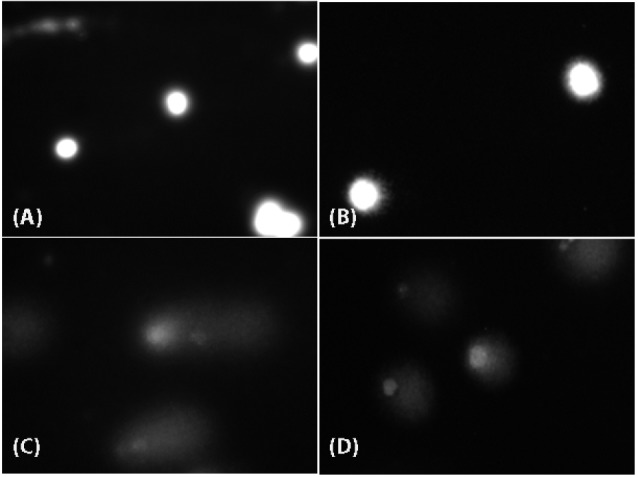
Evaluation of DNA damage potentials of CuO-NPs.
and (B) refer to negative (unexposed) HepG2 and Caco-2 cells, respectively. (C) and (D) refer to positive (100 µM H_2_O_2_) controls of HepG2 and Caco-2 cells, respectively.

The nanoparticles significantly induced DNA damage (1.2 to 9.6 -fold; *P* ≤ 0.05) (Figure 5). In the PC (100 µM H_2_O_2_) (Figure 6), tail intensity ranged from 13.2 ± 2.48 to 22.0 ± 2.11%. At the highest concentration (20 µg/mL), the tail intensities were 9.6 ± 1.01 and 41.8 ± 1.03%, for HepG2 and Caco-2 cells, respectively (approx. ≤9.6 -fold of NC). Caco-2 cells were more sensitive than HepG2 cells to CuO-NPs’ genotoxic potential (Figure 5; Figure 6).

### Oxidative damage

The nanoparticles induced oxidative damage was evaluated by assessing the cellular levels of GSH, MDA, 8-OHdG, and PC (Table 2). An increase in the levels of MDA (≤1.5-fold) and a decrease in the GSH levels (≤45.8%) were observed in HepG2 and Caco-2 cells. The increase in the MDA was significant in Caco-2 at concentrations >10 µg/mL, whereas the depletion in GSH levels were significant in both cell line at all exposure concentrations. On the other hand, the levels of PC and 8-OHdG protein and DNA oxidative damage biomarkers did not change significantly in both cell lines (≤1.63-fold).

**Table 2:** Evaluation of oxidative damage potentials of CuO-NPs

**Cells**	**Exposure concentration (µg/mL)**	**GSH (µmol /g protein)**	**MDA (µmol/g protein)**	**8-OHdG (µg/g protein)**	**PC (µg/g protein)**
HepG2	NC	53 ± 1.2	0.32 ± 0.05	1.1 ± 0.12	6.6 ± 0.57
	5	38 ± 0.99*	0.41 ± 0.03	0.91 ± 0.22	6.1 ± 0.64
	10	38 ± 1.1*	0.44 ± 0.01	0.97 ± 0.09	6.9 ± 0.52
	15	40 ± 0.95*	0.43 ± 0.05	1.1 ± 0.11	6.4 ± 0.49
	25	36 ± 1.4*	0.42 ± 0.03	1.1 ± 0.12	7.3 ± 0.62
Caco-2	NC	41 ± 1.3*	0.35 ± 0.02	0.96 ± 0.09	8.2 ± 0.35
	5	30 ± 0.86*	0.36 ± 0.05	0.99 ± 0.08	8.4 ± 0.35
	10	31 ± 0.63*	0.52 ± 0.04*	0.95 ± 0.15	8.1 ± 0.78
	15	27 ± 0.73*	0.51 ± 0.02*	0.96 ± 0.07	7.6 ± 0.48
	25	22 ± 0.57*	0.52 ± 0.04*	0.94 ± 0.09	8.4 ± 0.72

*Note*. The protein amount calculated for 4x10^4^ cells in every assay. The results were expressed as ± SD. * *P* ≤ 0.05 was significance. NC refers to negative control (unexposed cells).

### Apoptosis

**Figure 7:**
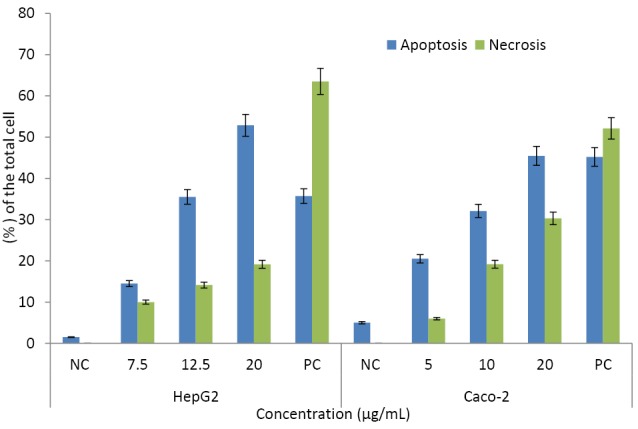
Evaluation on the apoptosis-inducing potentials of CuO-NPs.
The results were presented as percentage of the total cell amount ± SD. The unexposed cells were accepted as the negative control (NC), Cells incubated at 56ºC for 30 minutes used as positive control (PC) group.

The cell deaths in HepG2 and Caco-2 cells were highly induced by CuO-NPs. According to our results, apoptosis was seen to be the main cell death pathway in both cell lines. The apoptosis percentages were 52.9 ± 3.42% in HepG2 and 45.5 ± 4.86 % in Caco-2 cells. Necrosis was higher in Caco-2 cells as 30.3 ± 1.12 % of the total cells were positive for PI while this ratio was 19.1 ± 1.66% in the exposed HepG2 cells (Figure 7).

## Discussion

We evaluated to assess the toxicity profile of CuO-NPs (average size: 34.9 nm) in organs assume to be targeted for CuO-NPs after occupational or environmental exposure. It was observed CuO-NPs to take up by HepG2 and Caco-2 cells. By this means, the observed toxicity endpoints and morphological changes could be mainly due to CuO-NPs. Based on the Cu content in intracellular fluid, the cellular uptake potentials of CuO-NPs was higher in HepG2 than Caco-2 cells (Table 1). Cuillel et al reported CuO-NPs entered hepatic cells and bypassed the cellular defense mechanism against Cu. Also, the sub-toxic conditions of CuO-NPs induced a Cu overload and a Cu-Zn exchange on metallothioneins, and metal-regulatory transcription factor-1 regulation in both Cu and Zn homeostasis.^16^

Cellular sensitivity to cytotoxic damage induced by CuO-NPs was similar for HepG2 and Caco-2 cell lines (IC_50_: 10.04-12.19 µg/mL). Luo et al indicated CuO-NPs induced a decrease in viability, migration inhibition, G2/M phase cycle arrest, and especially mitogen-activated protein kinase activation in human keratinocytes and mouse embryonic fibroblasts.^27^ The cell viability of CuO-NPs decreased in mouse embryonic fibroblasts (48% at 10 µg/mL),^12^ and neuroblastoma (37% at 400 µg/mL) cells,^28^ in human lung epithelial (93% at 20 µg/cm^2^ and 50% at 15 µg/mL),^29,30^ airway epithelial (60% at 80 µg/cm^2^),^31^ alveolar adenocarcinomas epithelial (75% at 11 µg/mL),^32^ neuroblastoma (60%-70% at 0.01-10 µM), neuroglioma (25%-60% at 0.01-10 µM),^33^ C6 glioblastoma (10-1000 µM),^34^ cardiac microvascular endothelial,^15^ lymphocytes (50% at 0.04 mM),^35^ and colon cancer (50% at 40 µg/mL)cell lines.^36^ Muoth et al reported CuO-NPs caused a decrease in human chorionic gonadotropin release and microtissue viability in a 3D co-culture cell model of placental fibroblasts surrounded by a trophoblast cell.^37^ CuO-NPs induced cell membrane lysis, which leading to necrosis.^29,38^ Various combined parameters including endocytosis pathways, presence or absence of specific signaling receptors, mucus or glycocalyx could be affected the cytotoxic response of CuO-NPs in Caco-2 intestinal cells.^19^ Titma et al used Resazurin assay and TEER test to evaluate the changes in metabolic activity and permeability in Caco-2 and A549 cell lines (IC25 was 71 µg/mL).^32^ Viability of 48% at 10 µg/mL by Siddiqui et al and 4.69 mg/L as IC_50_ value by Wang et al were found in HepG2 cells exposed to CuO-NPs.^17,18^ Singh et al used HepG2 cell line as a model to evaluate their cytotoxic effect in textile fabrics, which be used CuO-NPs for being impregnated and reported a decrease of cell viability by 20-25% after 24 h.^39^

CuO-NPs induced DNA damage in all cells (1.2 - 9.6 -fold; *P* ≤0.05). CuO-NPs induced genotoxic responses via the p53 and p38 pathways in A549 lung cells.^11,30,38^ CuO-NPs significantly induced DNA damage at 12.5 µg/mL in mouse N2A neuroblastoma cells, with no decrease in cell viability.^28^ CuO-NPs induced both cell death and DNA damage in human A549 and BEAS-2B lung epithelial cells via disruption of cell membrane integrity.^40^ Akhtar et al observed the tail moment was 27% (5.4 fold compared to NC) at 15 µg/mL of CuO-NPs (*P* < 0.05).^41^

DNA damage significantly correlated with reactive oxygen species (ROS). However, there was no study on HepG2 and Caco-2 cell lines. It is well known that nanoparticles could cause depolarization and structural damage in mitochondria, leading to loss of mitochondrial membrane potential, the opening of the permeability transition pore, and increasing ROS and cell death.^42,43^ Similarly, it has been reported that the mechanism underlying CuO-NPs toxicity might be the induction of ROS generation or the oxidation of thiol groups by CuO-NPs that regulate pore status (open/close).^6,38,44,45^ Similarly, CuO-NPs damage with oxidative stress in HepG2 and Caco-2 cells (Table 2).

CuO-NPs induced oxidative damage in human keratinocytes,^46^ lymphocytes,^35^ hemocytes and gill cells,^47^ lungs epithelial,^1,11,29,41^ airway epithelial,^31^ and HepG2 cells,^17,19^ and in mouse BALB 3T3 embryonic fibroblasts.^12^ CuO-NPs caused up-regulation of plasminogen activator inhibitor-1 in mouse endothelial cells via oxidative stress.^48^ Canli et al reported an increase in the total oxidant status in the blood of rats exposed to CuO-NPs.^49^ Also, CuO-NPs were used as a chemical model for the induction of oxidative stress in rats to study the effectivity of resveratrol in the protection against oxidative damages in liver and kidney.^6^ In the present study, the decrease in cell viability observed could be due to an increase in oxidative stress after exposure to CuO-NPs (Table 2).

In our study, apoptosis was seen to be the main cell death pathway in HepG2 and Caco-2 cells. Similarly, CuO-NPs induced apoptosis in human MCF7 breast cancer^14^ multipotent mesenchymal stem and HepG2 cells.^17,50^ CuO-NPs induced apoptosis with an increase of caspase-3 levels in rats.^13^ CuO-NPs produced an abundance of transcripts coded for chemokine receptors, pro-inflammatory cytokines, or proteins in Caco-2 cells.^19^ Also, CuO-NPs induced apoptosis via a decrease in mitochondrial membrane potential with a concomitant increase in the gene expression ratio of Bax/Bcl2.^17^

## Conclusion

Some studies on CuO-NPs have shown a positive association with cytotoxicity, genotoxicity, apoptosis, and oxidative damage. However, the CuO-NPs’ toxicity on the liver and intestine are not clear yet. CuO-NPs could produce cellular toxic effects, as well as oxidative damage in the liver and intestine in vitro. The present study suggests that CuO-NPs should be carefully applied without being ignored their potential risk effects on human health.

## Ethical Issues

Not applicable.

## Conflict of Interest

The authors declared no conflict of interest.

## Acknowledgments

This work was supported by the Research Fund of Istanbul University (Project No: 42545).
